# Study on anti-inflammatory and immunomodulatory effects of clomipramine in carrageenan- and lipopolysaccharide-induced rat models of inflammation

**DOI:** 10.1080/13102818.2014.932136

**Published:** 2014-09-25

**Authors:** Ilia Kostadinov, Delian Delev, Atanaska Petrova, Irina Stanimirova, Krassimira Draganova, Ivanka Kostadinova, Marianna Murdjeva

**Affiliations:** ^a^ Department of Pharmacology and Clinical Pharmacology, Medical Faculty, Medical University-Plovdiv, Plovdiv, Bulgaria; ^b^ Department of Microbiology and Immunology, Faculty of Pharmacy, Medical University-Plovdiv, Plovdiv, Bulgaria

**Keywords:** clomipramine, carrageenan, lipopolysaccharide, inflammation, cytokines

## Abstract

The aim of the present study was to evaluate the anti-inflammatory effect of clomipramine in carrageenan- and lipopolysaccharide-induced (LPS-induced) models of inflammation by investigating the changes in serum levels of the pro-inflammatory cytokine TNF-α and the anti-inflammatory cytokines IL-10 and TGF-β after single and repeated administration of the drug.

In order to study the effect of single and repeated doses of clomipramine on carrageenan-induced paw oedema, male Wistar rats were divided in five groups (*n* = 8): control, positive control group and three experimental groups treated with 5, 10 and 20 mg/kg bw clomipramine, respectively. The effect of single and repeated doses of clomipramine on serum cytokine levels was studied as animals were divided in four groups: two control groups treated with saline and two experimental groups treated with clomipramine 20 mg/kg bw. Carrageenan and LPS were injected immediately after clomipramine or saline injection. Serum cytokine concentrations were tested by enzyme immunoassay.

Following acute administration only the highest dose that was used inhibited the carrageenan-induced inflammation. Oedema inhibition was observed with 5, 10 and 20 mg/kg bw clomipramine after repeated administration. Single and repeated administration of clomipramine at a dose of 20 mg/kg bw did not significantly change the serum levels of TGF-1β, IL-10 and TNF-α when compared to the controls in carrageenan-induced inflammation. Following LPS-induced inflammation clomipramine significantly increased the serum levels of TGF-1β after repeated administration and decreased TNF-α in rats after single-dose and repeated pretreatment with 20 mg/kg bw clomipramine. A significant increase in the levels of IL-10 in relation to this inflammatory model was observed only in single dose treated animals.

Clomipramine possesses an anti-inflammatory effect in the carrageenan-induced model of exudative inflammation. In LPS-induced inflammation, clomipramine showed an immunomodulatory effect, decreasing TNF-α and increasing TGF-1β after repeated administration, and increasing IL-10 after a single dose.

## Introduction

Growing evidence suggests that immune disregulation and inflammation may play a role in the pathophysiology of depressive disorders. According to the cytokine hypothesis depressive disorders are related to increased production of cytokines, including interleukins, tumour necrosis factor alpha (TNF-α) and interferon-α and -γ.[1] Higher blood levels of C-reactive protein, interleukin-6 (IL-6) and TNF-α are found in depressive patients compared to healthy subjects.[2] A meta-analysis of cytokines in major depression shows higher concentrations of the pro-inflammatory cytokines IL-6 and TNF-α in depressed patients compared with control subjects.[3]

The antidepressants amitriptyline and maprotiline exhibited anti-inflammatory activity in experimental conditions on carrageenan-induced model of inflammation in rats.[4–7] Abdel-Salam et al. [8] reported that amitriptyline, fluoxetine and trazodone showed anti-inflammatory activity in this model while sertraline exacerbated paw oedema. Fluoxetine exhibits anti-inflammatory effect in lipopolysaccharide (LPS)-stimulated microglial cell cultures through inhibiting the production of IL-6, TNF-α and nitric oxide,[9] and protects neurons against microglial activation-mediated neurotoxicity.[10]

Clomipramine is a tricyclic antidepressant which inhibits the reuptake of serotonin (5-hydroxytryptamine, hereafter 5-HT) and norepinephrine and interacts with some receptors such as histaminergic, cholinergic, adrenergic and 5-HT2 serotonergic.[11] Studies show that clomipramine inhibits interferon γ secretion and increases the synthesis of IL-10.[12,13]

IL-10 is a key regulator of depression symptoms and modulates depressive-like behaviour.[14] IL-10 knockout mice display increased depressive-like behaviour compared with the wild type and this is converted by injection of IL-10.[15] Therefore, the effect of clomipramine on the serum levels of this cytokine may contribute to its therapeutic effect in major depressive disorders. As IL-10 is an endogenous anti-inflammatory substance its up-regulation may also play role in the anti-inflammatory action of clomipramine.

TNF-α is a major inflammatory cytokine and depressed patients exhibit increased levels of this cytokine.[3] Moreover, it is produced by microglia and stimulates production of chemokines which attract immune cells to the damaged area in the brain.[16] The ability of clomipramine to reduce TNF-α plasma levels may also contribute to its therapeutic effect in depressive disorders.

Transforming growth factors (TGF) constitute a family of cytokines that promotes the induction of CD4+CD25+ T regulatory cells.[17] TGF-β inhibits both Th1 and Th2 reactions and plays a crucial role in supressing the immune system.

Carrageenan-induced paw oedema is a well-known model of inflammation for evaluation of anti-inflammatory activity of antidepressants. The carrageenan oedema is characterized by distinct phases with the involvement of different mediators. Release of nitric oxide and pro-inflammatory cytokines such as TNF-α and IL-1β are also involved in the delayed phase of carrageenan oedema.[6]

LPS from *Escherichia coli* cell wall is one of the most potent stimuli for cytokine release and is used as an experimental model for study the effects of antidepressants on the cytokine production.

The aim of the present study was to evaluate the anti-inflammatory effect of clomipramine in carragenan- and LPS-induced models of inflammation by investigating the changes in the serum levels of the pro-inflammatory cytokine TNF-α and anti-inflammatory cytokines IL-10 and TGF-β after single and repeated administration of the drug.

## Materials and methods

### Animals

Male Wistar rats with average weight of 220–250 g were used. Animals were housed under standard laboratory conditions: 12:12 hours light/dark cycle, room temperature 26.5 °C ± 1 °C, and free access to food and water. Experiments were performed between 8:00 am and 3:00 pm

### Chemicals

Λ-carrageenan (Sigma-Aldrich GmbH), clomipramine hydrochloride (Novartis Pharma AG, Switzerland), diclofenac sodium (Hexal AG, Germany) and LPS from *E. coli O55* (Sigma-Aldrich GmbH) were used. Carrageenan was dissolved in isotonic saline and 1% solution was used. Clomipramine hydrichloride was dissolved in isotonic solution and an emulsion was prepared using Tween 20. TGF-1β, IL-10 and TNF-α Platinum ELISA kits (eBioscience, Austria) for rats were used for measurement of serum cytokine levels.

### Carrageenan-induced paw oedema

Paw oedema was induced by injecting 100 μl of a 1% solution of Λ-carrageenan in saline into right hind paw of the rat. Hind paw volume was measured immediately before carrageenan injection and at the 2nd, 3rd, 4th and 24th hour thereafter with a pletismometer (Ugo Basile, Italy).

### LPS-induced inflammation

LPS was dissolved in isotonic saline and was injected intraperitoneally in dose 250 μg/kg bw four hours before blood collection.

### Experimental design

The effect of a single dose i.p. clomipramine on carrageenan-induced paw oedema was studied in the first series of experiments. Animals were divided in five groups (*n* = 8). The control group received only saline, the positive control group was treated with diclofenac sodium 25 mg/kg bw and the three experimental groups were treated with 5, 10 and 20 mg/kg bw clomipramine, respectively. Paw volume was measured prior to carrageenan injection and at the 2nd, 3rd, 4th and 24th hour after in order to determine the difference in the paw volume.

The effect of repeated doses i.p. clomipramine on carrageenan-induced paw oedema was studied in the second series of experiments. The experimental animals, the control and positive control groups were treated as previously described in the first series of experiments but the treatment lasted 14 days and carrageenan oedema was induced on day 15. Paw volume was measured as described in the first series of experiments.

The effect of a single dose i.p. clomipramine on serum cytokine levels was studied in the third series of experiments. Animals were divided in four groups: two control groups treated with saline and two experimental groups treated with clomipramine 20 mg/kg bw. Carrageenan and LPS were injected immediately after clomipramine or saline injection and blood samples were collected 4 hours thereafter.

The effect of repeated doses i.p. clomipramine on serum cytokine levels was studied in the fourth series of experiments. Animals were divided in four groups as described in the third series of experiments but were treated for 14 days. Carrageenan and LPS were injected on day 15 immediately after clomipramine or saline injection and blood samples were collected 4 hours thereafter.

### Measurement of serum cytokine levels

TGF-1β, IL-10 and TNF-α concentrations were measured in diluted serum samples from rats collected 4 hours after carrageenan and LPS injection using solid-phase ELISA. Assays were performed according to the manufacturer's instructions. Absorbance was read at 450 and 620 nm using an ELISA reader. Absorbance was then recalculated as a concentration (pg/ml) using a standard curve. The detection limits of the employed assays were as follows: TGF-1β - 8 pg/ml, IL-10 – 1.5 pg/ml and TNF-α – 11 pg/ml. Intra-assay and inter-assay reproducibility varied as follows: for TGF-1β <3.7% and < 8.6%; for IL-10 <5% and <10%; for TNF-α <5% and <10%.

### Statistical analysis

Data were analysed using the independent-sample *t*-test from the software product SPSS 11.0. Mean values (*X*) ± SEM were calculated. Results were considered significant at *p* < 0.05.

## Results and discussion

### Effect of acute administration of clomipramine on carrageenan-induced paw oedema

**Figure 1.**
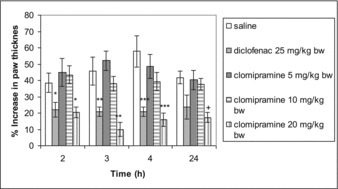
Anti-inflammatory effect of clomipramine in carrageenan-induced paw oedema after single administration. *p < 0.05 compared with saline in the 2nd hour; **p < 0.05 compared with saline in the 3rd hour; ***p < 0.05 compared with saline in the 4th hour; + *p* < 0.05 compared with saline in the 24th hour.

Clomipramine at a dose of 5 and 10 mg/kg bw i.p. did not show significant anti-inflammatory effect when compared with the control. The highest dose that was used caused significant inhibition in the development of paw oedema on the 2nd, 3rd, 4th and 24th hour (*р* = 0.02; *р* = 0.003; *р* < 0.0001, *р* < 0.0001) as compared to the control group. The reference drug diclofenac caused significant inhibition of oedema on the second, third and fourth hour (*р* = 0.043; *p* = 0.022 and *р* = 0.006) post-carrageenan challenge (Figure 1).

### Effect of repeated administration of clomipramine on carrageenan-induced paw oedema

**Figure 2.**
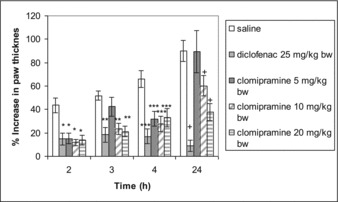
Anti-inflammatory effect of clomipramine in carrageenan-induced paw oedema after repeated administration. *p < 0.05 compared with saline in the 2nd hour; **p < 0.05 compared with saline in the 3rd hour; ***p < 0.05 compared with saline in the 4th hour; + *p* < 0.05 compared with saline in the 24th hour.

Clomipramine at a dose of 5 mg/kg significantly inhibited paw oedema only on the second (*р* = 0.002) and fourth hour (*р* = 0.003) when compared with the control. Doses of 10 and 20 mg/kg bw clomipramine produced a significant anti-inflammatory effect on the 2nd (*p* = 0.001 and *р* = 0.002, respectively), 3rd (*р* < 0.0001 for both groups), 4th (*р* = 0.002 and *p* = 0.008, respectively) and 24th hour (*р* = 0.037 and *р* = 0.001, respectively) post-carrageenan as compared to the control group. The reference drug diclofenac caused significant inhibition of oedema on all tested hours (*р* = 0.004; *р* = 0.002; *р* < 0.0001 and *р* < 0.0001) (Figure 2).

Tricyclic antidepressants have been shown to possess anti-inflammatory activity in experimental models of inflammation. Clomipramine is a tricyclic drug that non-selectively inhibits norepinephrine and 5-HT reuptake. In experimental conditions clomipramine has been reported to decrease the inflammation induced by yeast or carrageenan injection in the rat hind paw.[8,18,19] Our results are in agreement with these data. In addition anti-inflammatory activity in our carrageenan model of inflammation was demonstrated after repeated administration. The intimate mechanism of this effect is not fully understood. Romero et al. [20] have shown that clomipramine given i.p. at 10 mg/kg bw increased cortical 5-HT levels. The doses used in our study are higher than those that have been shown to produce an increase in the brain levels of 5-HT in rats. Serotonin mediates anti-inflammatory activity in the central nervous system (CNS). Intracerebroventricular injection in experimental conditions of exogenic 5-HT on rats with normal serotonin levels reduces carrageenan oedema.[21] Serotonin releasing substances like amphetamine supress immune functions.[22] The anti-inflammatory effect of serotonin in CNS can be explained with its neuroendocrine action. *In situ* hybridization on rat brain slices with oligopeptides showed an increase of corticotropin releasing hormone mRNA in the paraventricular nucleus and proopiomelanocortin in the anterior pituitary lobe upon stimulation of 5-HT receptors.[23] It can be suggested that anti-inflammatory effect of clomipramine is due to depletion of intracellular 5-HT, and increased extracellular 5-HT levels in the CNS.

Maes et al. [12] have summarized that 5-HT has a negative immunoregulatory effect as indicated: 5-HT decreases mitogen-induced T lymphoproliferative responses; supresses lymphocyte DNA synthesis; inhibits the migration of mononuclear leukocytes and T-cell activation of normal spleen cells; decreases INF-γ-induced major histocompatibility antigen class II expression on macrophages and the synthesis of TNF-α by macrophages. Therefore anti-inflammatory action of clomipramine that was observed in our study can be partially explained with its ability to increase the serotonin levels in the immune system. T-lymphocytes express high-affinity 5-HT transporter and it is potently inhibited by clomipramine.[24]

### Effect of i.p. clomipramine 20 mg/kg bw on the serum levels of the anti-inflammatory cytokines TGF-1β and IL-10

Single and repeated i.p. administration of clomipramine at dose 20 mg/kg bw in carrageenan-induced inflammation did not significantly change the serum levels of TGF-1β and IL-10 when compared with the controls.

**Figure 3.**
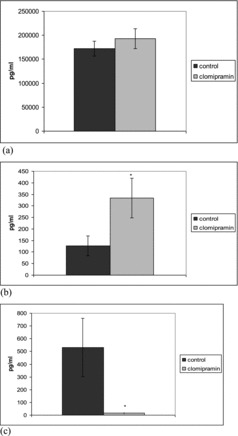
Effect of single-dose clomipramine treatment on serum levels of TGF-β (a), IL-10 (b) and TNF-α (c) in rats challenged with LPS. *p < 0.05 compared with saline.

**Figure 4.**
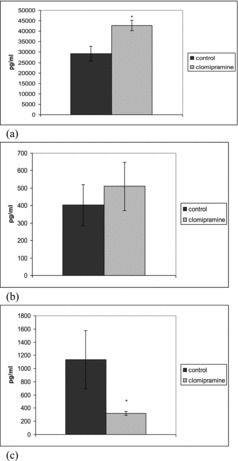
Effect of repeated clomipramine treatment on serum levels of TGF-β (a), IL-10 (b) and TNF-α (c) in rats challenged with LPS. *p < 0.05 compared with saline.

Clomipramine significantly increased the serum levels of TGF-1β in LPS-induced inflammation after repeated administration. In this inflammatory model significant increase in the levels of IL-10 was observed only in single dose treated animals (Figures 3 and 4).

It is established that TGF-1β showed lower values in patients with major depression than in healthy controls.[25,26] Plasma TGF-1β levels were significantly increased after 8-week treatment. In our study a two-week pretreatment with clomipramine significantly increased the serum TGF-1β in LPS-induced inflammation. After single-dose pretreatment, clomipramine also tended to increase the levels of TGF-1β in this model of inflammation, but the results did not reach statistical significance. TGF-1β has a crucial role in the generation of CD8+ T suppressor cells that reduce the antibody production and also induces CD4+CD25 + regulatory T cells which inhibit T-cell responses.[17] Clomipramine may suppress inflammation and change the pro-inflammatory/anti-inflammatory cytokine balance via increased TGF-1β.

IL-10 is one of the most important anti-inflammatory cytokines. It inhibits the production of pro-inflammatory cytokines such as TNF-α and IL-6.[27] Clomipramine significantly increases serum IL-10 levels in LPS-induced inflammation after single-dose administration and non-significantly from repeated one. Ohgi et al. [28] found that 5-HT plays an important role in IL-10 upregulation, increasing the serum levels of IL-10 in mice treated with LPS. It is likely that stimulatory effect of clomipramine on IL-10 production is due to increased serotonin levels. IL-10 is an endogenous anti-inflammatory substance that is produced by both T-cells and monocytes/macrophages. [29] Diamond et al. [13] have demonstrated that the ability of low doses of antidepressants to increase IL-10 production occurs as a result of increased T-cell derived production, as opposite to monocyte-derived IL-10. In addition data indicate that IL-10 plays role in mediating the suppressive effects of antidepressants on INF-γ, as IL-10 is not increased by the doses of antidepressants that suppress INF-γ production. Based on the known anti-inflammatory actions of IL-10 our results show that this cytokine may contribute to the observed anti-inflammatory action of clomipramine in LPS-induced inflammation. Clomipramine also tends to increase IL-10 production in the carrageenan model of inflammation but this effect failed to reach statistical significance. This may be due to the limited number of samples and the large degree of inter-individual variability. Our study is the first showing the stimulating effect of clomipramine on the production of IL-10 using an *in vivo* model for LPS stimulation.

### Effect of i.p. clomipramine 20 mg/kg bw on the serum levels of the pro-inflammatory cytokine TNF-α

LPS increased the TNF-α level in control rats. The levels of TNF-α in rats treated with a single dose and repeatedly pretreated with 20 mg/kg bw clomipramine were significantly reduced in comparison to the animals that were treated with saline after the LPS challenge (Figures 3 and 4). Clomipramine treatment failed to alter production of the pro-inflammatory cytokine TNF-α in response to carrageenan.

Clinical data demonstrate that serum concentration of TNF-α is increased in patients with major depressive disorder when compared with healthy controls.[2] Antidepressant treatment diminished its levels.[26,30] In experimental conditions, clomipramine, imipramine and citalopram strongly inhibit the release of this cytokine from monocytes after 10 hours of incubation in the presence of LPS.[31] In vivo studies on rats showed that amitriptyline decreased the TNF-α concentration into the carrageenan-injected paw tissues after systemic and central administration.[6] In our study intraplantar injection of carrageenan increased the plasma levels of TNF-α only in chronically treated rats and although clomipramine decreased its levels the result did not reach statistical significance. As it was expected LPS increased the levels of TNF-α in saline treated groups. Our results indicate that clomipramine decreased TNF-α after LPS treatment. Ohgy et al. [28] found that pretreatment with SSRIs (selective serotonin reuptake inhibitors), SNRIs (selective norepinephrine reuptake inhibitors) and 5-HT attenuated LPS-induced increase in TNF-α and they increased IL-10 serum levels in mice. The mechanism through which the antidepressants diminish the production of this pro-inflammatory cytokine remains unclear. In LPS stimulated microglial cultures, antidepressants that selectively block the reuptake of serotonin and norepinephrine reduced TNF-α production after four hour incubation. When cells were incubated with serotonin or norepinephrine, a significant reduction in LPS-induced TNF-α production was observed.[32] As clomipramine increases the levels of both serotonin and norepinephrine, this may contribute to its ability to reduce TNF-α plasma concentrations. Kubera et al. [33] showed that in whole blood cultures stimulated with LPS and PHA extracellular 5-HT in concentrations above the baseline physiological levels supress the production of IL-6 and TNF-α. Other authors found that 5-HT in a broad spectrum of concentrations between 10^−10^ M and 10^−7^ M inhibited LPS-induced TNF-α synthesis. Durk et al. [34] found that serotonin receptor subtypes 4 and 7 are expressed in monocytes and their stimulation decreases LPS-induced TNF-α release.

## Conclusions

The findings of the present study showed that clomipramine possesses an anti-inflammatory effect in a carrageenan-induced model of exudative inflammation after single and repeated administration. This action may be useful in the treatment of some inflammatory conditions. In LPS-induced inflammation, clomipramine showed an immunomodulatory effect. It decreased the levels of the pro-inflammatory cytokine TNF-α after single and repeated administration. An increased level of the anti-inflammatory cytokine IL-10 was observed after a single dose, while TGF-1β levels were increased after repeated administration of clomipramine. 
